# Dual-channel high-speed functional photoacoustic microscopy with ultra-wide field of view

**DOI:** 10.1038/s41377-025-02114-3

**Published:** 2026-01-28

**Authors:** Van Tu Nguyen, Carlos Taboada, Jesse Delia, Tri Vu, Luca Menozzi, Soon-Woo Cho, Jing Li, Nishad Jayasundara, Anthony DiSpirito, Junjie Yao

**Affiliations:** 1 Department of Biomedical Engineering, Duke University, Durham, NC 27708 USA; 2 Biology Department, Duke University, Durham, NC 27708 USA; 3 Department of Biological Sciences, Vanderbilt University, Nashville, TN 37232 USA; 4 Nicholas School of the Environment, Duke University, Durham, NC 27713 USA

**Keywords:** Imaging and sensing, Scanning probe microscopy

## Abstract

Photoacoustic microscopy (PAM) systems often face challenges in simultaneously achieving high speed, high resolution, high sensitivity, and a large field of view (FOV). To address this challenge, we have developed dual-channel PAM (DC-PAM) that can expand the FOV without compromising the imaging speed, detection sensitivity, or spatial resolution. DC-PAM has two identical, independent channels of laser excitation and acoustic detection. It exploits two facets of a single hexagon scanner to concurrently steer the dual excitation laser beams and the resultant acoustic waves. DC-PAM achieves an ultra-wide FOV of 22.5 × 24 mm² with a total functional imaging time of ~15 s. Proof-of-concept experiments were conducted using DC-PAM on freely-swimming zebrafish, hypoxia-challenged mice, and sleeping glassfrogs, all of which benefit from the large FOV and high imaging speed to track the dynamic and physiological processes at the whole-organ or whole-body level. These applications demonstrate the potential of DC-PAM for a wide range of biological studies.

## Abstract (web summary)

Dual-channel photoacoustic microscopy (DC-PAM) enables ultra-wide-field, high-speed, and high-resolution imaging, advancing whole-organ and whole-body studies in freely moving or physiologically active animals.

## Graphical abstract

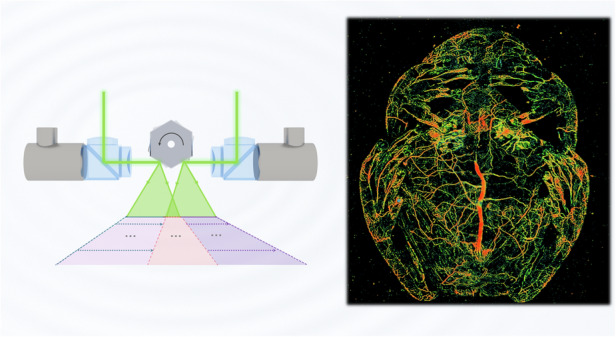

## Introduction

Over the past decade, advancements in optical imaging technologies have transformed life sciences. High-resolution optical imaging provides fine structural details and physiological activities in tissues^1–4^. However, achieving high-resolution imaging often comes at the expense of a limited field of view (FOV) or imaging speed^5^. For dynamic imaging applications, such as imaging freely moving organisms, a wide FOV is highly desirable. As an optical imaging technology, photoacoustic microscopy (PAM) enables volumetric imaging by detecting acoustic signals generated through the photoacoustic effect^6–8^. More importantly, PAM offers functional^9^ and molecular imaging^10^ of a large range of endogenous and exogenous contrasts, such as measuring oxygenation^11,12^, blood flow^13,14^, and oxygen metabolism^15–17^.

However, like other optical imaging techniques, PAM faces the technical challenge of simultaneously achieving a high imaging speed, large FOV, high spatial resolution, and high detection sensitivity^18^. Traditional PAM systems rely on slow motorized scanning^19,20^, but recent advancements in high-speed PAM have introduced faster scanning mechanisms, such as piezo scanners^21^, slider crank scanners^22,23^, galvo scanners^24–27^, micro-electromechanical systems (MEMS) scanners^8,28–31^, and polygon scanners^32,33^. Among them, the polygon scanner stands out by offering high-speed, repeated line scanning across a large range^34^. It holds an advantage over galvo and MEMS scanners, as its scanning range is independent of the scanning speed^35–37^. However, the ultrasound detector in high-speed PAM often limits the FOV or compromises detection sensitivity. These limitations hinder the large-scale imaging tasks, such as whole-body imaging and simultaneous imaging of multiple organisms.

To enhance the performance of high-speed PAM, we introduce a dual-channel PAM (DC-PAM) system equipped with two independent imaging channels. A single water-immersible hexagon scanner is used to steer two laser beams and their resultant PA signals at the same time. This unique design is enabled by the symmetric geometry of the hexagon scanner. Two facets of the hexagon are used simultaneously, resulting in a line scan that is twice as wide as that in previous polygon-based PAM systems^38–40^. We have demonstrated that DC-PAM can achieve an ultra-wide FOV of 22.5 × 24 mm², with a total imaging time of 15 seconds. Using dual-wavelength excitation, we have validated that DC-PAM can simultaneously image the hemodynamic responses in two mice, one under normoxia and the other under hypoxia. Furthermore, we have shown that DC-PAM can map the functional dynamics of glassfrog transparency at the whole-body level and track the group behavior of freely-swimming zebrafish.

## Results

### Dual-channel PAM

**Fig. 1:**
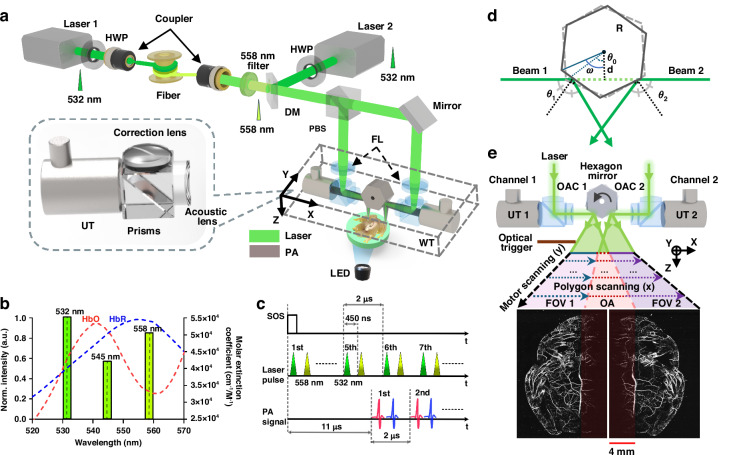
The principle of DC-PAM. **a** Schematic of the DC-PAM system. HWP half-wave plate, PBS polarization beamsplitter, DM dichroic mirror, FL focusing lens, WT water tank, UT ultrasound transducer, LED light emitting diode. **b** The spectrum of the Raman-shifter output, and the absorption spectra of oxy-hemoglobin (HbO_2_) and deoxy-hemoglobin (HbR). **c** The dual-wavelength excitation sequence and the resultant PA signals from HbO_2_ and HbR. SOS, start of scan. **d** The optical scanning in DC-PAM. R radius of polygon; d distance from the center of polygon to the laser beam; θ_0_ the initial angle of polygon; θ_1_ the angle between polygon and the laser beam 1; θ_2_ the angle between polygon and the laser beam 2. **e** Scheme of DC-PAM volumetric scanning, which is achieved by fast polygon scanning along the x-axis (i.e., fast axis) and stepper motor scanning along the y-axis (i.e., slow axis). UT ultrasound transducer, OAC optical-acoustic combiner, FOV field of view, OA overlapping area

The overview of the DC-PAM system is illustrated in Fig. 1a. Two pulsed lasers (SPFL-532-40, Spectra-Physics) are employed as the pump sources. A half-wave plate is placed after the laser output to adjust the polarization of the laser beams. One laser beam is coupled into a 25-m-long single-mode fiber (SMF-28, Thorlabs) to generate the 558-nm light via the stimulated Raman scattering (SRS) effect (Fig. 1b). A bandpass filter (560-nm central wavelength, 10-nm bandwidth, model #87-887, Edmund Optics Inc.) is used to pass the 558-nm wavelength. The other 532-nm beam is merged with the 558-nm beam using a dichroic mirror (T550lpxr-UF1, Chroma Technology Corp). The 558-nm laser pulse lags the 532-nm pulse by 450 ns (Fig. 1c). The merged beams are split into two sub-beams by a polarization beamsplitter (CCM5-PBS201, Thorlabs).

Figure 1e shows the compact design of the DC-PAM imaging head. The two optical excitation sub-beams are delivered to two optical-acoustic combiners (OAC), which are placed on the opposite sides of the polygon scanner. The OAC includes a correction lens, a focusing lens, two right-angled prisms, a 30 MHz ultrasound transducer (V213-BC-RM, Olympus), and an acoustic lens. The detected PA signals are amplified (AML0040-00-6010, Wenteq Microwave) with a gain of 60 dB before being digitized at 250 MHz (ATS9350, AlazarTech). No signal averaging is performed. The detailed structure of the DC-PAM imaging head is shown in Fig. 1a and Fig. S1. To protect electrical components, including the polygon scanner, we sealed the imaging head in a 3D-printed chamber with a shaft and ball bearings at both ends to ensure smooth scanning. Each polygon facet is 7.0 × 10 mm², providing a 14-mm scanning range per channel. The overlapping area is ~4 mm near the edge of each scanning range, resulting in a total effective scanning range of 24 mm^39^.

Each laser pulse generates a one-dimensional time-resolved PA signal, and volumetric imaging is achieved through fast polygon scanning along the *x*-axis (fast axis) and stepper motor scanning along the *y*-axis (slow axis) (Fig. 1e). To synchronize the fast-axis with the slow-axis, a multimode fiber is mounted at the beginning of the line scanning to provide a “start of scan” signal. Since the time interval between two laser pulses is shorter than the PA signal travel time from the target to the ultrasound transducer, we precisely synchronize the laser firing and data acquisition with the scanning (Fig. S5). We use a 450-ns interval between the dual-wavelength laser pulses at 532 and 558 nm (Fig. 1c), which corresponds to a spatial separation of approximately 0.675 mm in soft tissue with a speed of sound of 1500 m/s. This spatial separation is larger than the optical depth of focus (~0.221 mm), avoiding overlapping between the two-wavelength PA signals within a single A-line for accurate spectral unmixing, even for a slightly uneven tissue surface (Fig. 1b).

The dual-beam steering by the polygon scanner (namely Channel 1 and Channel 2) is illustrated in Fig. 1d, Supplementary Video 1, and Fig. S1. The steering angles of the two channels follows:

$$\left\{\begin{array}{c}{\theta }_{1}+{\theta }_{2}={60}^{0}\left({60}^{0}-{\theta }_{0} < \omega < {\theta }_{0}\right)\\ {\theta }_{1}+{\theta }_{2}={120}^{0}\left(\omega \ge {\theta }_{0}\right)\end{array}\right.$$

Where *R* is the radius of the polygon; *d* is the orthogonal distance between the incident laser beam and the center of polygon; ${\theta }_{0}{=\cos }^{-1}(\frac{d}{R})$ is the initial angle of the polygon; *θ*_1_ and *θ*_2_ are the incident angle of the laser beam relative to the polygon facet, respectively for Channel 1 and Channel 2; *ω* is the scanning angle of the polygon. There is practically no interference between the two channels. Although an overlapped scanning region may occur when the sum of *θ*_1_ and *θ*_2_ is 60°, the optical foci of the two channels are designed such that their respective depths of field do not overlap, and thus the resultant PA signals effectively do not interfere. Furthermore, each channel uses an independent ultrasound transducer, which, combined with the spatial separation of the optical foci, efficiently prevents PA signal interference between the channels (Fig. S1).

For phantom experiments, DC-PAM can achieve a maximum time-resolved A-line rate of 2 MHz at 532 nm and a B-scan frame rate of 500 Hz (Fig. S3d). For in vivo experiments, we usually use a laser repetition rate of 500 kHz, a B-scan frame rate of 300 Hz, and an average scanning step size of 8.4 μm. This adjustment is to minimize the potential photothermal damage to the tissue over prolonged imaging sessions^41^. The lateral resolution and axial resolution are estimated to be 7.5 and 33 μm, respectively (Fig. S2). With a step size of 5 μm for the slow axis and a B-scan rate of 300 Hz for the fast axis, the total scanning time for an FOV of 22.5 mm × 24 mm is ~15 s. The distortion of the images due to the rotatory scanning has been corrected by using a depth correction method^42^. For each channel, the optical excitation beam was co-axially aligned with the acoustic detection axis of the ultrasound transducer to maximize the signal-to-noise ratio (SNR). To minimize inter-channel misalignment during simultaneous scanning, the dual-channel probe housing was fabricated using high-precision CNC machining. Given that the axial resolution of the ultrasound transducer is ~33 µm, the machining precision introduces negligible alignment error. When inter-channel misalignment does occur due to mechanical drifting, image registration techniques can be applied in post-processing to correct for spatial offsets.

**Fig. 2:**
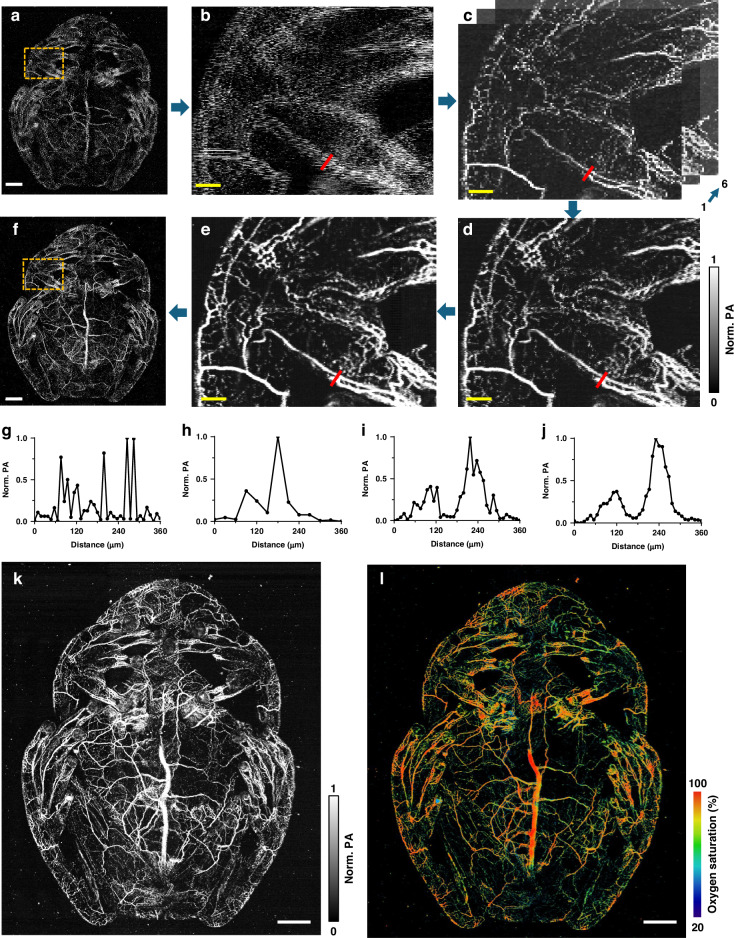
Image registration and deep-learning-based upsampling. **a** The misaligned and undersampled PA image of the glassfrog vessels, assembled from all six facets of the polygon scanner. **b** Close-up image of the dashed box region in (**a**), showing the misalignment of facets. **c** Close-up images generated by every facet. **d** Close-up assembled image after deep-learning-based upsampling. **e** Close-up assembled image after facet alignment. **f** The whole-body glassfrog image after facet alignment and deep-learning upsampling. **g**–**j** The intensity profiles of the representative vessels marked by the red lines in (**b**–**e**). **k** The vasculature image of an awake glassfrog with facet alignment and upsampling. **l** The oxygen saturation image of the same glassfrog. Scale bar in (**a**, **f**, **k**, **l**), 1 mm. Scale bar in (**b**–**e**), 500 μm

Figure 2 and Fig. S4 show the data processing workflow, including facet registration, deep-learning-based upsampling, and frame stabilization. The polygon scanner immersed in water suffers from the wobbling motion due to the water damping force, along with minor size variations among its six facets. This results in misaligned scanning trajectories and distorted images (Fig. 2a, b). Each facet can scan consistently (Fig. 2c), but the large step size and undersampling lower the image quality. To address these issues, we have developed a geometric-transformation registration method, which aligns scanning paths of different facets to correct facet misalignment. Applying this across all facets improves image quality (Fig. 2d, e).

Based on our previous work summarized in the Supplementary Information^40^, we utilized a modified FD U-net model to upsample the registered DC-PAM images. The model was trained using the high-resolution PAM images of mouse brain vasculature^43^, which were numerically downsampled to simulate undersampling. This deep-learning-based upsampling method corrects the undersampling without causing blurring or jagged edges like traditional interpolation methods, and avoids creating spurious fake structures (i.e., “hallucinations”)^44–46^. It complements our facet alignment by removing minor misalignment artifacts and improves image quality without increasing the laser pulse repetition rate or risking the thermal damage (Fig. 2f). For further evaluation, Fig. 2g–j illustrate that the enhancement in vessel structures contributes to improved quantitative analysis. Figure 2k, l displays final DC-PAM images of an awake glassfrog, showing the whole-body vasculature network and vessel-by-vessel sO_2_ map.

### Simultaneous functional imaging of two mice

**Fig. 3:**
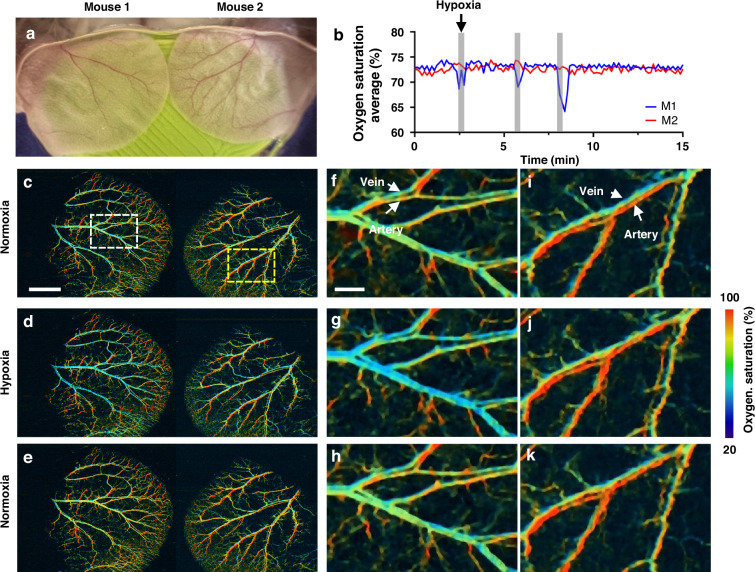
Functional DC-PAM of hypoxia challenge on two mice. **a** Photograph of the right ear of Mouse 1 and the left ear of Mouse 2 positioned on the same imaging platform. Mouse 1 was under hypoxia treatment and Mouse 2 was the normoxia control. **b** The average sO_2_ changes in the two mouse ears. **c**–**e** sO_2_ images of the two mouse ears at three time points indicated in (**b**). **f**–**h** Close-up sO_2_ images of Mouse 1 ear as indicated by the dashed white boxes in (**c**–**e**). **i,** **j**, **k** Close-up sO_2_ images of Mouse 2 ear as indicated by the dashed yellow boxes in (**c,** **d**, **e**). Scale bar in (**c**, **d**, **e**), 2 mm. Scale bar in (**f**–**k**), 500 μm

In preclinical research, studying two mice simultaneously, with one serving as a control and the other undergoing treatment, allows for direct comparative analysis of physiological responses under the same experimental conditions. This approach minimizes inter-subject variability caused by environmental fluctuations or instrument drift, thereby improving the reliability of functional imaging data. By leveraging DC-PAM’s ultra-wide field of view, we imaged the ears of two mice simultaneously, with one mouse (namely Mouse 1, or M1) undergoing three cycles of hypoxia challenge and the other mouse (namely Mouse 2, or M2) serving as a control (Fig. 3a). Figure 3c–e present the sO_2_ maps of the mouse ears under normoxia and hypoxia challenge. At baseline (normoxia), there was a clear difference in sO_2_ levels between the arteries (shown in red, indicating higher HbO_2_ levels) and veins (shown in blue, indicating higher HbR levels). However, during the hypoxia challenge, the fraction of HbR increased, and the sO_2_ levels became more uniform across the vessels. DC-PAM tracked the dynamics of three hypoxia cycles, as shown in Fig. 3b and Supplementary Video 2.

### Freely-swimming zebrafish

**Fig. 4:**
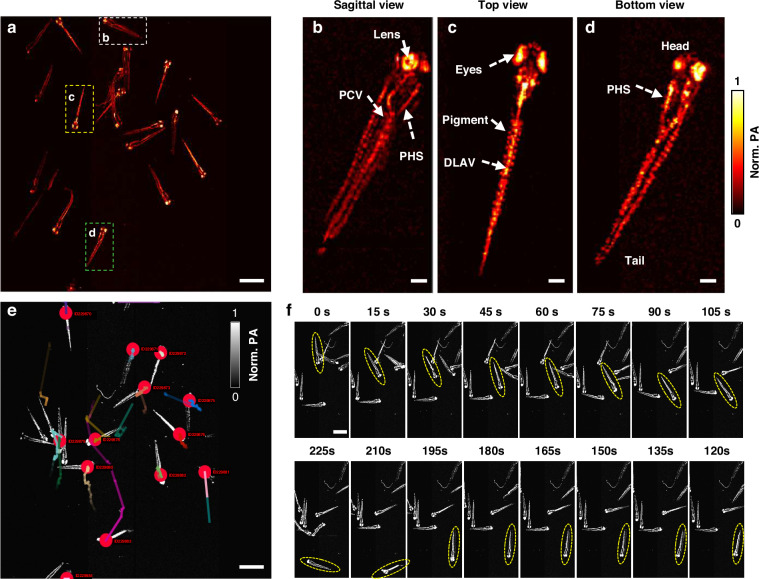
DC-PAM of freely-swimming zebrafish. **a** DC-PAM image of multiple zebrafish larvae. **b**–**d** Close-up images of three representative zebra fish indicated by the dash boxes in (**a**), corresponding to sagittal, top, and bottom views of the zebrafish. **e** Tracking the movement of the zebrafish over 120 s. **f** Serial PA images of swimming zebrafish. The dashed ovals highlight the track of a single fish. DLAV dorsal longitudinal anastomotic vessel, PCV posterior cardinal vein, PHS primary head sinus. Scale bar in (**a**, **e**), 2 mm. Scale bar in (**b**–**d**), 250 μm. Scale bar in (**f**), 1.5 mm

Zebrafish is a popular animal model for toxicological studies, developmental biology, and drug screening, due to their genetic similarity to humans and their transparent bodies during early stages, which allow for direct imaging of internal structures. Studying freely-swimming zebrafish provides a powerful approach to investigating physiological dynamics in a natural, unrestrained condition. Here, we applied DC-PAM to record zebrafish larvae freely swimming in a 22.5 mm × 24 mm open arena. Figure 4a shows the images of anesthetized 8-day post-fertilization (dpf) fish. From the close-up images (Fig. 4b–d), we clearly observed anatomical structures of zebrafish, such as the retinal pigment epithelium of the eye, body pigments, anterior and posterior cardinal veins, primary head sinus, and the dorsal longitudinal anastomotic vein. We tracked 15 fish using Trackmate ImageJ, an open-source tracking algorithm (Supplementary Video 3), over a total period of 900 seconds. The high throughput of DC-PAM allowed us to observe fine details over a very wide FOV, capturing the dynamic swimming events, as shown in Fig. 4e and Supplementary Video 4. Unlike traditional PAM methods that require immobilization, DC-PAM provides the real-time observation of zebrafish development in response to environmental or physiological stimulation, and contributes to our understanding of their cardiovascular function, metabolic regulation, and group behavior in relatively natural conditions.

### Glassfrog (*Hyalinobatrachium fleischmanni*)

**Fig. 5:**
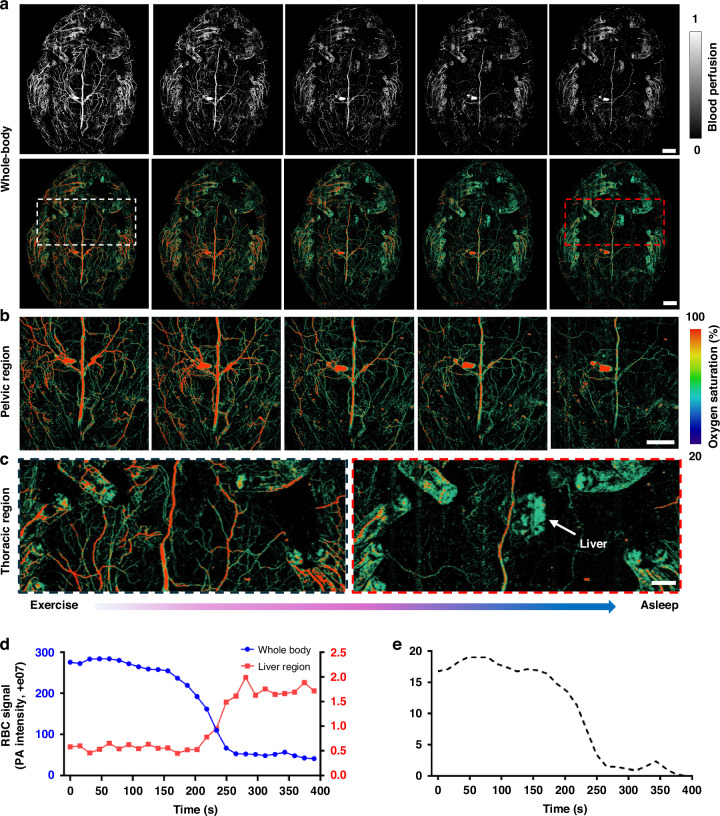
DC-PAM of glassfrog hemodynamics when transitioning from exercise to sleep. **a** A glassfrog was imaged at multiple time points following induced exercise until it resumed sleeping. Whole-body images were acquired to measure blood perfusion (top) and sO_2_ changes (bottom). Note the gradual decrease in systemic perfusion, the decrease in sO_2_, and the increase in liver perfusion, as the frog transitioned from exercise to sleep. **b** Close-up sO_2_ images of the pelvic area. **c** Close-up images of thoracic area are indicated by the dashed boxes in (**a**). **d** Quantitative changes in blood perfusion over the whole body and liver region during the transition from exercise to sleep. **e** Quantitative changes in whole-body sO_2_. Scale bar for (**a**, **b**), 2 mm. Scale bar for (**c**), 1 mm

Studying the hemodynamics of glassfrogs provides unique insights into their extraordinary ability to regulate blood perfusion and maintain transparency during sleep, a rare physiological adaptation with potential biomedical implications^1^. Understanding these hemodynamic mechanisms not only sheds light on the evolutionary strategies of glassfrog transparency but also offers a comparative model for studying circulatory adaptations, blood pooling mechanisms, and potential applications such as thrombosis, vascular disorders, and microcirculatory regulation. Here, we used DC-PAM to conduct functional whole-body imaging of glassfrogs to document their mechanism for removing most red blood cells from the circulation by sequestering them in their liver during sleep (Fig. 5 and Supplementary Video 5). Figure 5 shows whole-body blood perfusion and sO_2_ images at different time points as the glassfrog resumed sleep after exercise stimulation. Blood perfusion, along with sO_2_, gradually decreased, while the signal from their liver increased (as shown in Fig. 5d), confirming the findings that glassfrogs can hide their red blood cells in the liver to maintain transparency during sleep^1^.

**Fig. 6:**
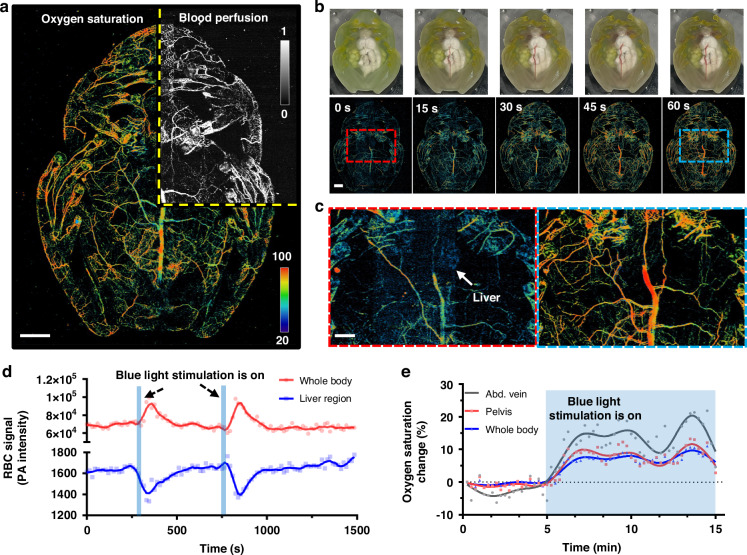
DC-PAM of glassfrog hemodynamic changes in response to light stimulation. **a** Composite DC-PAM images of blood perfusion and sO_2_ of an awake glassfrog. **b** Serial photograph and sO_2_ images of a glassfrog after blue light stimulation. **c** Close-up sO_2_ images of the dashed boxes in (**b**). Note that the liver signals decreased after the blue light stimulation. **d** PA signal change in the liver region and non-liver region during two cycles of blue light stimulation. **e** Quantitative changes in sO_2_ in the abdominal vein and pelvic area, and on the whole-body level, in response to the blue light stimulation. Scale bar for (**a**, **b**), 2 mm. Scale bar for (**c**), 1 mm

We further investigated the glassfrog’s response to blue light stimulation, which is known to relax blood vessels in vertebrates^47^. Figure 6a shows the DC-PAM image of an awake female glassfrog. Photographs and corresponding sO_2_ images illustrate increased blood perfusion in the systemic circulation (Fig. 6b) and decreased liver signals (Fig. 6c) upon blue light stimulation. These results indicate that blue light can act as a stimulus that induces the release of red blood cells from the liver back to the systemic circulation, suggesting a similar mechanism to the transition from exercise to sleep (Fig. 5). To evaluate the reversibility of the hemodynamic changes induced by blue-light stimulation, we quantified blood perfusion in the liver region and the whole-body non-liver region during two 30-s stimulations, as shown in Fig. 6d. The results suggest a reversible mechanism of pooling and release of red blood cells in and out of the liver. To further investigate the changes in sO_2_ induced by blue light, we imaged a glassfrog during a 10-min stimulation event (Fig. 6e and Supplementary Video 6) and quantified the sO2 values in the abdominal vein, pelvic area, and the entire body. In all cases, sO_2_ increased by ~7–20% over the baseline. These results highlight the dynamic and reversible physiological changes induced by blue light stimulation, which may resemble the frog’s natural transition from between sleeping and awakening.

## Discussion

In summary, our dual-channel photoacoustic microscopy (DC-PAM) system achieves a balanced combination of ultra-wide field of view (22.5 × 24 mm²), high-speed functional imaging (B-scan rate >500 Hz), and high spatial resolution (~7.5 µm lateral resolution and ~33 µm axial resolution). DC-PAM is a useful functional imaging technology for large-scale imaging applications. DC-PAM leverages a water-immersible polygon scanner that concurrently steers two independent excitation laser beams and the resultant ultrasound waves. Consequently, DC-PAM allows for high-throughput, high-fidelity imaging of whole biological systems, as demonstrated in zebrafish, mice, and glassfrogs. Particularly, while our previously reported polygon-based PAM was used to image only the abdominal region of the glassfrogs^1^, DC-PAM was capable of imaging the whole-body glassfrogs at a high speed. This improvement allowed for a detailed study of the frog’s hemodynamic changes as they transitioned from exercise to sleep. The results align well with our previous study, which demonstrated that glassfrogs concealed their blood in the liver during sleep while maintaining their transparency^1^. Additionally, we have observed that blue light can reversibly increase glassfrog’s systemic perfusion in a manner similar to the process of awakening, and this systemic perfusion coincides with a decrease in the red blood cells inside the liver, suggesting an interesting mechanism of pooling and releasing red blood cells. In these studies, the expanded FOV not only improves the imaging throughput but also enables new biological insights that would be challenging to study using conventional PAM systems. These results collectively demonstrate that DC-PAM provides a promising method to investigate functional, dynamic, and physiological changes on different small animal models.

However, the current DC-PAM system presents certain limitations. Firstly, our setup requires water immersion of the polygon scanner, which significantly constrains both the maximum imaging speed and image quality due to the damping force and resistance of water. In contrast, polygon scanners operating in air offer greater stability and faster scanning than their water-immersed counterparts. To address this issue, cylindrically focused ultrasound transducers with a large detection aperture ( > 20 mm) coupled with rapid optical scanning can be employed to eliminate the need for water immersion. Second, the current system relies on a motorized scanning stage to translate the sample, which limits the volumetric imaging rate. To address this, a faster linear motorized stage could be implemented to improve the scanning speed. However, while this solution may increase the volumetric imaging rate, translating the sample at a high-speed presents challenges for in vivo experiments. Therefore, further optimization is needed to balance the scanning speed with the need for stable imaging for in vivo applications. Third, the current dual-channel configuration has a 4-mm overlapping area, ~28% of the single-channel scanning range. This overlapping is primarily resultant from the hexagon scanner’s geometry and large scanning angle. It reduces the effective total scanning range, as some laser pulses are wasted to illuminate the overlapped scanning range. To mitigate this issue, an octagon scanner can be explored to provide dual-beam steering. Compared with the hexagon scanner, the octagon scanner, with its larger number of facets, facilitates smoother transitions between facets and reduces the scanning angle, which can improve the frame rate and expand the effective field of view. The octagon mirror reduces the scanning angle to approximately 90 degrees, leading to reduced beam path overlapping between channels. Importantly, this design does not require shortening the transducer’s focal length and thus would not sacrifice the image quality.

In conclusion, the improved imaging capabilities of DC-PAM open new avenues for studying dynamic physiological processes in vivo with a large FOV and a high speed, paving the way for future applications in neurovascular research, developmental biology, oxygen metabolism, cardiovascular disease, and pharmacological screening. Further advancements in scanning stability, real-time image processing, and integration with complementary imaging modalities will further expand DC-PAM’s potential in preclinical research.

## Materials and methods

### Animals

All animal experiments were conducted in compliance with Institutional Animal Care and Use Committee (IACUC) guidelines at Duke University (Protocols: A009-20-01, A174-2108, and A069-22-04-25) and followed NIH guidelines for the care and use of laboratory animals.

Mice: The optical pulse energy at the ear surface was ~200 nJ per wavelength, with laser beams focused 250 μm beneath the skin surface. Glassfrogs: The optical pulse energy used for glassfrog imaging was ~250 nJ per wavelength. Zebrafish: The optical pulse energy used for zebrafish imaging was ~50 nJ per wavelength.

### Hexagon scanner

The six-facet hexagon scanner was fabricated from BK-7 glass with aluminum-coated optical facets for high reflectivity (>90% at 532 nm). It features a clear facet aperture of 7 mm × 10 mm along the fast and slow scanning axes, respectively, and a polygon diameter of 14 mm. The hexagon scanner is powered by a direct-current electric motor (M1N10FB11G, Minebea Mitsumi), enabling smooth operation at speeds up to 10,000 rpm, corresponding to a B-scan rate of 1 kHz.

### SRS-based dual-wavelength laser system

The stimulated Raman scattering (SRS)-based dual-wavelength laser system was constructed using a 25-m single-mode fiber (SMF-28, Thorlabs) aligned on a fiber alignment stage (561D-XYZ, Newport) with a bare fiber chuck (FPH-S, Newport). The fiber output was secured with a universal bare fiber terminator (BFT1, Thorlabs) and collimated via an achromatic fiber port (PAF2A-A10A, Thorlabs). The output laser spectrum was analyzed using an optical spectrometer (CCS175, Thorlabs).

### Systemic hypoxia in mice

BALB/c mice (3–5 months old, male, 20–30 g) were used for in vivo imaging of the hypoxia challenge. To induce systemic hypoxia in mice, a 3D-printed nose cone was used to deliver regulated oxygen levels while maintaining anesthesia with 1.5% v/v isoflurane. Body temperature was regulated at 37 °C using a heating pad. Hypoxia was induced by reducing the oxygen concentration from 21 to 3% using flowmeters. The mice were first imaged under normoxic for 2 min, followed by a 30-s hypoxia (or stay under normoxia for the control mouse) exposure, with this cycle repeated three times.

### Glassfrog experiments

Glassfrogs (Hyalinobatrachium fleischmanni) were housed in bioactive vivariums that mimicked their natural habitat, maintained at 22–25 °C, ≥70% relative humidity, and a 12-h light/dark cycle with frequent misting. They were fed a varied diet of crickets and fruit flies every 2 to 4 days. Two experimental assays were conducted:

Exercise-induced hemodynamic changes: Frogs were gently encouraged to jump within a plastic bucket for 5–10 min by tapping their posteriors. They were then transferred to Petri dishes and immediately imaged to capture the hemodynamic recovery process as they transitioned to a resting state.

Blue light-induced hemodynamic changes: Blue light exposure (455 nm, M455L4, Thorlabs) was used to assess its effect on vasodilation and systemic blood perfusion. Glassfrogs, known to concentrate red blood cells (RBCs) in their hepatic sinusoids while sleeping, were imaged before and after blue light stimulation to track RBC redistribution and circulatory dynamics.

### Zebrafish experiments

Wild-type zebrafish (Ekkwill strain) were imaged at 8 and 14 days post-fertilization (dpf). The fish were housed in a recirculating AHAB system (Aquatic Habitats, Apopka, FL) at 28 °C under a 14-h light/10-h dark cycle. Larvae were bred in Danieau’s embryo medium and anesthetized using Tricaine Methanesulfonate (MS222, 0.01%) at pH 7.2 to slow down movement during imaging. This method effectively reduces motion without long-term harm, as previously described^48^.

## Supplementary information

Supplemental material

Supplementary Movie 1

Supplementary Movie 2

Supplementary Movie 3

Supplementary Movie 4

Supplementary Movie 5

Supplementary Movie 6

## Data Availability

The data that support the findings of this study are available from the corresponding author upon reasonable request.
