# Supplementary material for: Dual-channel high-speed functional photoacoustic microscopy with ultra-wide field of view
Source: Light Sci Appl. 2026 Jan 28;15:91. doi: 10.1038/s41377-025-02114-3 (PMC12852836; doi:10.1038/s41377-025-02114-3)
Supplement: Supplementary file 1 — Supplemental material [file 41377_2025_2114_MOESM1_ESM.docx]

**Supplementary information for**

**Dual-channel High-Speed Functional Photoacoustic Microscopy with Ultra-wide Field of View**Authors: Van Tu Nguyen^1,†^, Carlos Taboada^1,2,3,†,*^, Jesse Delia^3^, Tri Vu^1^, Luca Menozzi^1^, Soon-Woo Cho^1^, Jing Li^1^, Nishad Jayasundara^4^, Anthony DiSpirito^1^, Junjie Yao^1,*^

^1^Department of Biomedical Engineering, Duke University, Durham, NC 27708, USA.

^2^Biology Department, Duke University, Durham, NC 27708, USA.

^3^Department of Biological Sciences, Vanderbilt University, Nashville, TN 37232, USA.

^4^Nicholas School of the Environment, Duke University, Durham, NC 27713, USA.

^†^These authors contributed equally to this work.

^*^Corresponding author: [carlos.taboada@duke.edu](mailto:carlos.taboada@duke.edu) and [junjie.yao@duke.edu](mailto:junjie.yao@duke.edu)

**
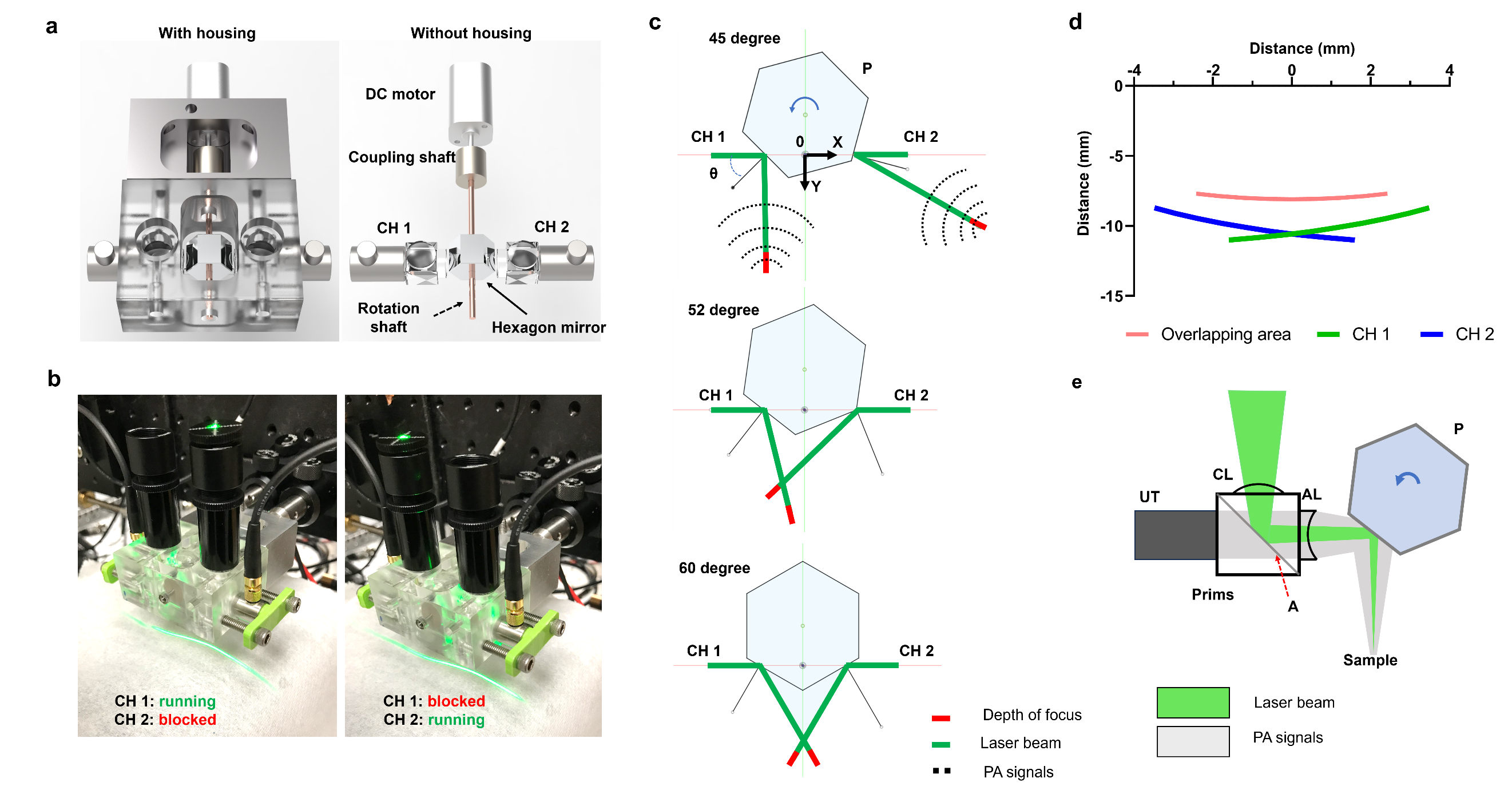
**

**Fig. S1. DC-PAM system.** (**a**) The detailed design of the compact DC-PAM imaging head with and without the 3D-printed housing. (**b**) Photograph of the working DC-PAM system for each channel. (**c**) Steering of the laser beams of two channels at three different polygon scanning angles. (**d**) Plot of the focal points and the corresponding overlapping area of the two channels. (**e**) The detailed components of OAC. P, polygon mirror. UT, ultrasound transducer. CL, correction lens. AL, acoustic lens. A, aluminium layer.

**Fig. S1** illustrates the detailed design of the DC-PAM system, showing each laser beam during operation. The beam overlapping region is located at the center of the DC-PAM probe and occurs only when each laser beam is transitioning between two adjacent facets. As shown in **Fig. S1c**, the optical focal zones lie outside the overlapping region. As illustrated in **Fig. S1d**, the overlapping path of the two beams is highlighted in red, while their respective focal points, shown by the green and blue lines, are much deeper. This spatial separation ensures that no detectable signal interference occurs between the two channels.

Each optical-acoustic combiner (OAC), depicted in **Fig. S1e**, consists of two right-angle prisms, an acoustic lens, and a correction lens. The prisms are glued at their hypotenuse surfaces, which are coated with a thin aluminium layer. This coating reflects the laser light while transmitting the ultrasound waves, enabling efficient optical-acoustic path combination. The acoustic lens converts spherical photoacoustic waves into planar waves to match the flat ultrasound transducer. The correction lens compensates for optical aberrations introduced by the prism and acoustic lens, improving the laser beam’s focusing.


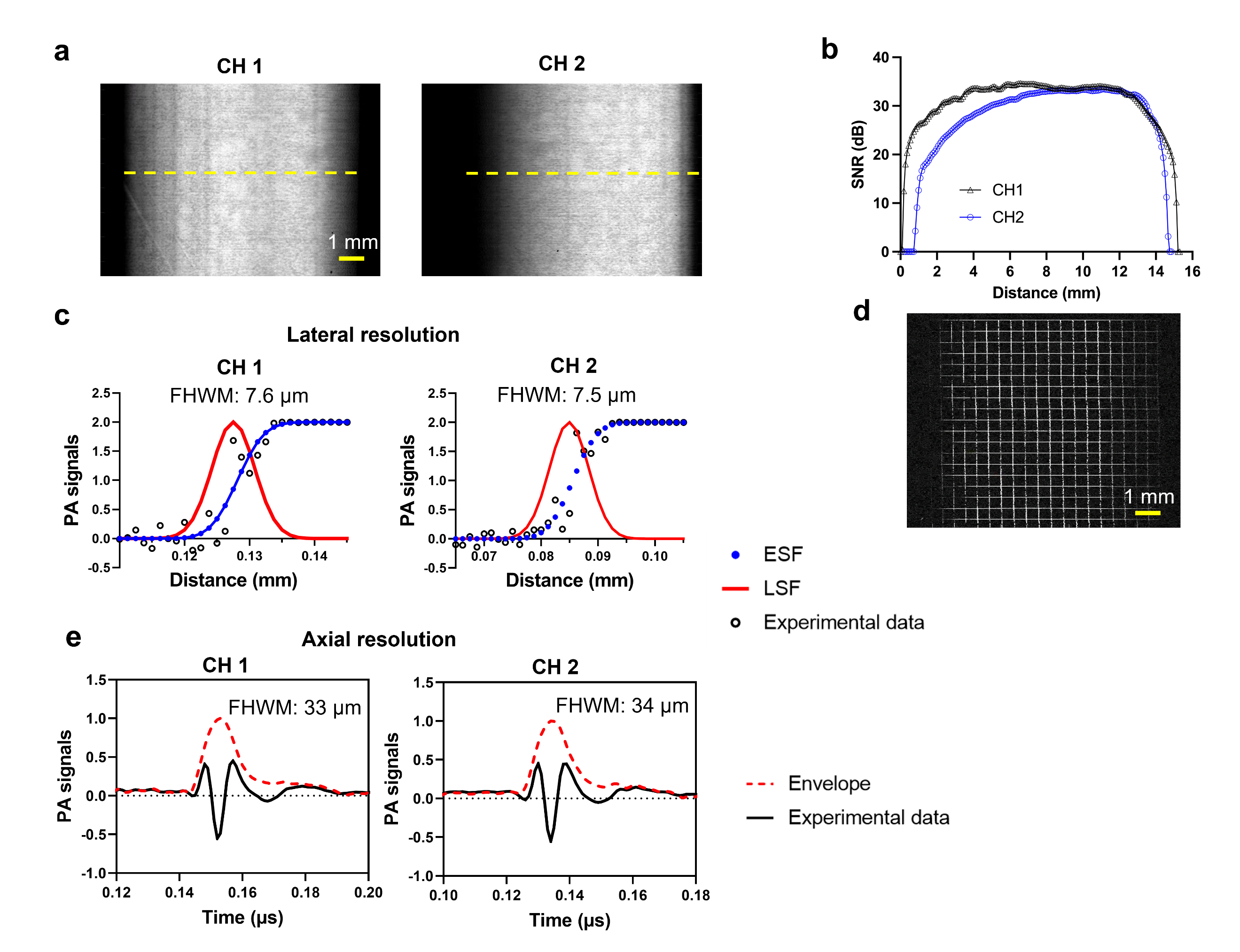


**Fig. S2. Characterization of DC-PAM. (a)** Measurement of the sensitivity of Channel 1 and 2 by imaging a uniform black tape. (**b)** Signal-to-noise ratio (SNR) along the dash line in (**a**). (**c)** Quantification of the lateral resolution by imaging a resolution target. ESF, edge spread function; LSF, line spread function. (**d)** Image of a distortion target. (**e**) Quantification of the axial resolution by imaging a resolution target.

**
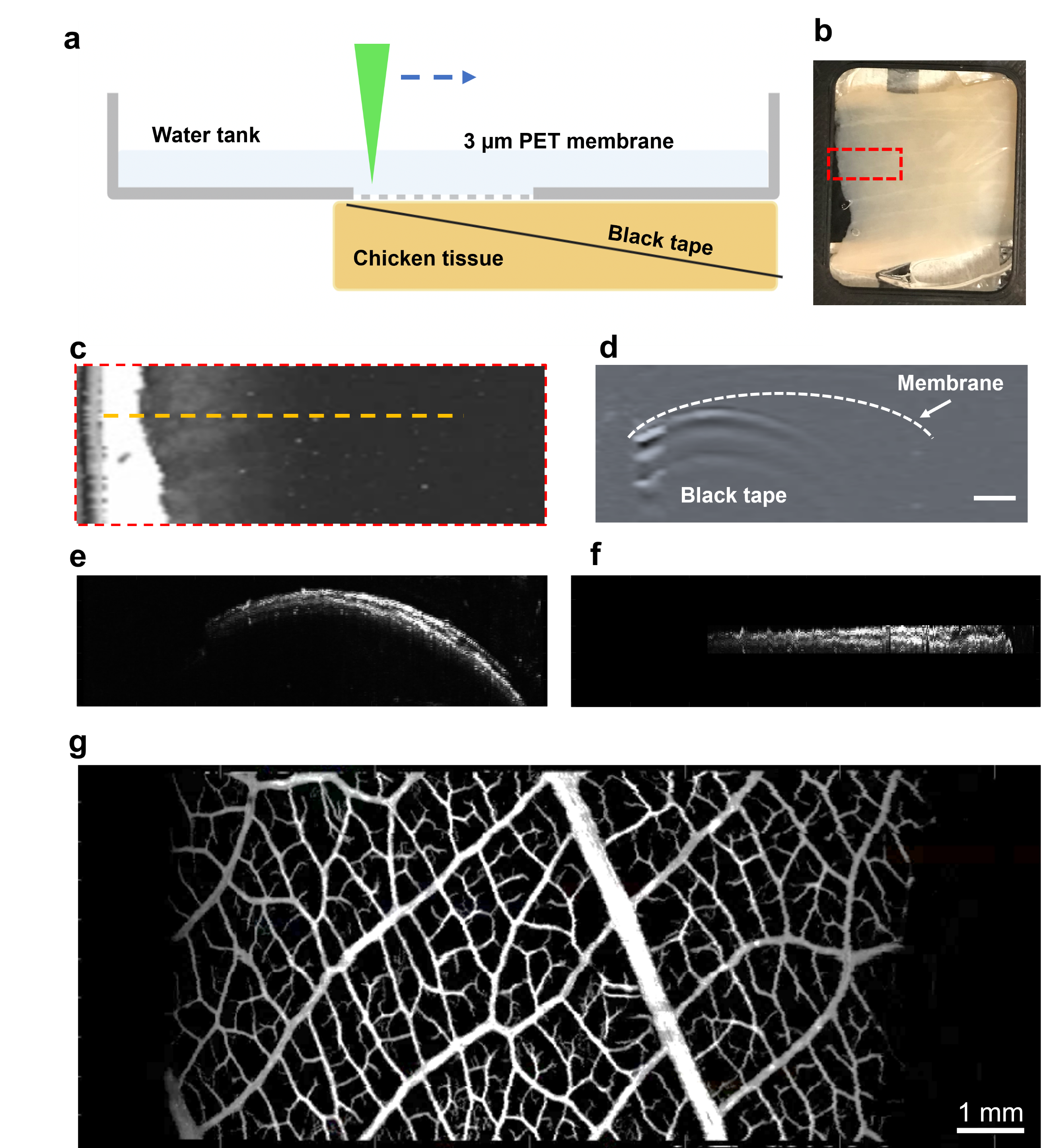
**

**Fig. S3. Imaging depth and correction in DC-PAM.** (**a**) Experimental setup to estimate the imaging depth. (**b**) The photograph of sample, which is black tape inserted into fresh chicken breast tissue. (**c**) Photoacoustic image of the dashed box region in (**b**). (**d**) Cros-sectional image along the dashed line in (**c)**. (**e-f**) Cross-sectional images before and after depth correction. (**g**) Skeleton leaf imaging with laser frequency of 2 MHz and B-scan rate of 500 Hz by DC-PAM system. Scale bar: 2 mm for (**c-f**).


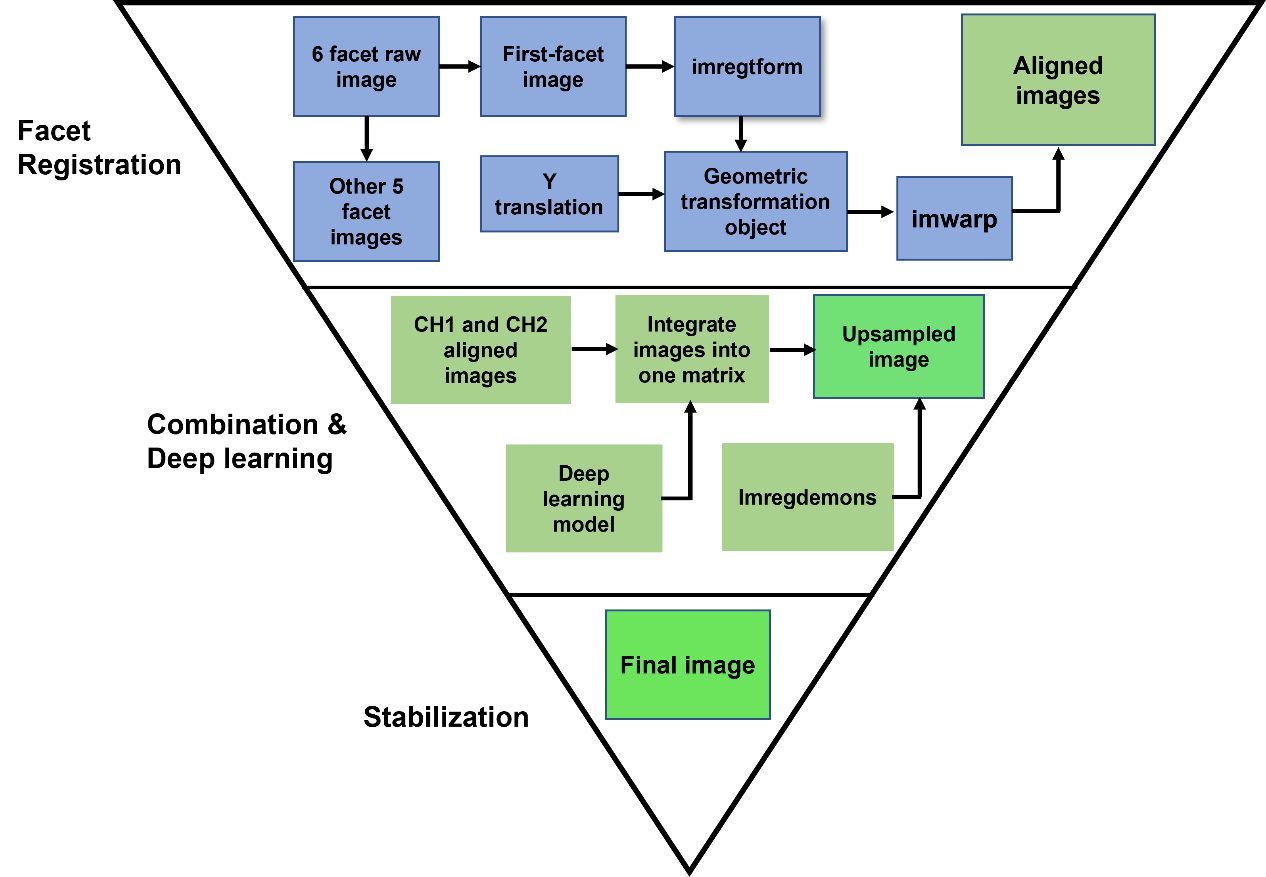


**Fig. S4. The workflow of facet registration and deep-learning-based upsampling.**

Our FD U-net architecture previously reported in [40], a U-net variant incorporating dense blocks for improved feature discrimination and training efficiency, includes three key modifications: (1) ELU activation replaces ReLU to improve training stability and speed in deeper networks; (2) Max pooling is replaced with a learned downsampling scheme using 1×1 and 3×3 convolutions with stride 2; (3) 2D spatial dropout (p = 0.05) is introduced within each convolution block to enhance generalization, convergence, and reduce model uncertainty. The model was trained on the Duke PAM dataset [43]; a dataset of 381 high-resolution PAM images of mouse brain vasculature, acquired using a traditional high-resolution PAM system. Fully-sampled images were numerically downsampled to simulate various levels of undersampling; for example, a 2:1 downsampling in the *x*-direction drops every second column. To restore image dimensions, zero-filling was applied, effectively masking missing/undersampled pixels with zeros. Images were then zero-padded to sizes divisible with 128, randomly cropped to 128×128 patches, and augmented with random rotations, shearing, intensity shifts, Gaussian noise, and JPEG compression artifacts. Training was conducted over 200 epochs using the Adam optimizer (learning rate of 0.005), with 10 random crops per training and validation image. Model checkpoints were selected based on validation loss. The loss function combined pixel-wise and Fourier-domain MSE to promote accuracy in both spatial and frequency domains, along with a perceptual term based on SSIM and PSNR. The Fourier and perceptual losses in particular serve to penalize over-reconstructed, spurious vessels, since randomly hallucinated features are unlikely to exhibit the frequency structure or spatial coherence expected of correctly upsampled vascular patterns produced from uniformly downsampled inputs.


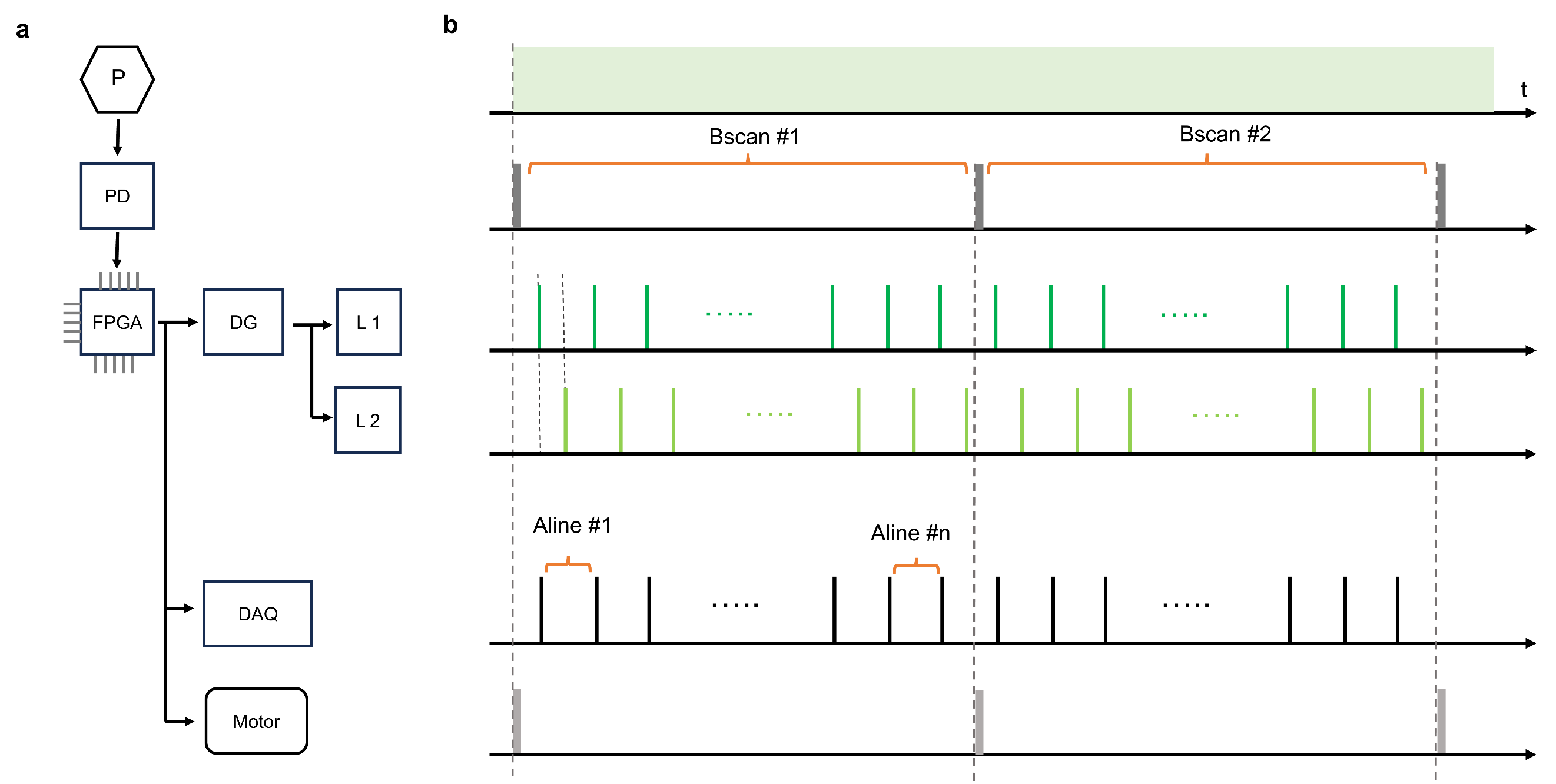


**Fig. S5. Synchronization of the DC-PAM system. (a)** Trigger flow that synchronizes the key system components. (**b)** Triggering sequence. P, polygon mirror. PD, photodiode. FPGA, field-programmable gate array. DG, delay generator. L1: laser 1. L2, laser 2. DAQ, data acquisition system.

As shown in **Fig. S5**, the polygon begins rotating at a stable speed. The lasers operate in two modes: arm mode and fire mode, configured with external trigger. Laser light in the arm mode is used to trigger the photodiode (PD) when a new facet is engaged in steering the laser beam (the fast axis). This PD signal serves as the start-of-scan (SOS) trigger for the field-programmable gate array (FPGA). Once triggered, the FPGA sends synchronized trigger pulses to the lasers, data acquisition system (DAQ), and the motorized scanning stage (the slow axis). Each SOS signal initiates a B-scan, while each laser trigger initiates an A-line. Simultaneously, the motorized scanning stage advances by one step after each B-scan and continues moving until the desired number of B-scans is reached to form a C-scan. Once the C-scan is completed, the motorized scanning stage reverses its direction to begin the next C-scan. A delay generator provides a 450 ns delay between Laser 1 and Laser 2.

**Supplementary Movie Captions**

**Supplementary Movie 1.** The working principle of DC-PAM, showing the hexagon scanner steering two laser beams simultaneously.

**Supplementary Movie 2.** DC-PAM of two mouse ears at the same time, with M1 as the control under normoxia and M2 challenged by hypoxia for 3 cycles.

**Supplementary Movie 3.** DC-PAM of several freely-swimming zebrafish at 8 dpf (left),with motion tracking via TrackMate (right).

**Supplementary Movie 4.** DC-PAM of freely-swimming zebrafish at 14 dpf (top), with depth encoded in color from blue (deep) to red (shallow) (bottom).

**Supplementary Movie 5.** DC-PAM of hemodynamic changes in glassfrogs transitioning from exercise to sleep, including the blood perfusion (left) and oxygenation (right).

**Supplementary Movie 6.** DC-PAM of hemodynamic changes in glassfrog in response to blue light stimulation, including the blood perfusion (left) and oxygenation (right).
